# The Nation

**Published:** 1867-02

**Authors:** 


THE NATION—A Weekly Journal containing Literary, Artistic, and Scientific Intelligence, Criticisms of Books, Pictures, and Music, Foreign Correspondence, and Deliberate Comments on the Political and Social Topics of the Day.

It is published every Thursday in New York, in quarto form, of twenty pages, and of very attractive typography. It is now in the second year of its existence, and has already earned for itself higher encomiums than were ever before bestowed on any American journal.

Published by E. L. Godkin & Co., 130 Nassau street, New York.

TERMS—$5 per annum, $3 for six months, in advance.

